# Senolytics Reduce Coronavirus-Related Mortality in Old Mice

**DOI:** 10.1093/geroni/igab046.951

**Published:** 2021-12-17

**Authors:** Christina Camell

**Affiliations:** Institute on the Biology of Aging and Metabolism, University of Minnesota, Province, Minnesota, United States

## Abstract

The elderly and chronically ill are among groups at the highest risk for morbidity and mortality to several infections, including SARs-CoV-2. Cellular senescence contributes to inflammation, multiple chronic diseases, and age-related dysfunction, but effects on responses to viral infection are unclear. Old mice acutely infected with pathogens that included a SARS-CoV-2-related mouse β-coronavirus experienced increased senescence and inflammation with nearly 100% mortality. Targeting SCs using senolytic drugs before or after pathogen exposure significantly reduced mortality, cellular senescence, and inflammatory markers and increased anti-viral antibodies. Thus, reducing the SC burden in diseased or aged individuals should enhance resilience and reduce mortality following viral infection, including SARS-CoV-2.

